# Een cohortvergelijking van eetgedrag, leefstijl en sociaal-emotionele gezondheid bij jongeren vóór en tijdens de coronacrisis

**DOI:** 10.1007/s12508-022-00331-4

**Published:** 2022-03-08

**Authors:** Nina van den Broek, Maaike Verhagen, Junilla Larsen, Jacqueline Vink

**Affiliations:** Behavioural Science Institute, Radboud Universiteit, Nijmegen, Nederland

**Keywords:** coronacrisis, jongeren, eetgedrag, leefstijl, sociaal-emotionele gezondheid, Corona crisis, Adolescents, Food intake, Lifestyle, Social-emotional health

## Abstract

De coronacrisis kan beschouwd worden als een ‘natuurlijk experiment’ waarin de sociale context aanzienlijk is veranderd. Het is nog grotendeels onbekend hoe jongeren daarop reageren en welke gedragingen veranderen. We hebben een cohortvergelijking gemaakt van derdeklassers (*N* = 177, *M*_leeftijd_ = 15,00) van de middelbare school vóór de coronacrisis (voorjaar 2019) met een qua demografische kenmerken vergelijkbare groep derdeklassers (*N* = 188, *M*_leeftijd_ = 14,95) tijdens de eerste lockdown (voorjaar 2020). We vergeleken de domeinen eetgedrag, leefstijl en sociaal-emotionele gezondheid tussen beide cohorten en keken naar de samenhang binnen en tussen domeinen voor beide cohorten. De consumptie van zoete snacks thuis, alcoholgebruik en eenzaamheid waren in het coronacohort hoger dan vóór de coronacrisis (niet significant na correctie). De consumptie van fruit thuis, van suikerhoudende dranken en ongezonde snacks buitenshuis, en matige en zware fysieke activiteit kwamen minder voor tijdens de lockdown. Relatietevredenheid met ouders, geluksgevoel en stress door school waren lager vergeleken met het cohort vóór de lockdown. Roken, stress thuis en relatietevredenheid met beste vriend(in) waren in beide cohorten even hoog. Het patroon van samenhang binnen en tussen de domeinen verschilde. Verder onderzoek moet uitwijzen hoe veerkrachtig jongeren op de langere termijn zijn en welke jongeren ondersteuning nodig hebben om terug te veren.

**Digitaal aanvullende content:**

De online versie van dit artikel (10.1007/s12508-022-00331-4) bevat aanvullend materiaal, toegankelijk voor daartoe geautoriseerde gebruikers.

## Abstract (en)

The corona crisis can be considered a ‘natural experiment’, in which the social context has changed dramatically. It is largely unknown how adolescents respond to this, and which behaviors change. We have compared a cohort of ninth graders (*N* = 177, *M*_age_ = 15.00) from secondary schools before the corona crisis (Spring 2019) with a demographically comparable group of ninth graders (*N* = 188, *M*_age_ = 14.95) during the first lockdown (Spring 2020). We compared the domains food intake, lifestyle, and social-emotional health between both cohorts, and we examined the associations within and between the domains for both cohorts. Sweet snack intake from home, alcohol use, and loneliness were higher in the corona cohort compared to before the corona crisis (non-significant after correction). The intake of fruit from home, of sugar-sweetened beverages and unhealthy snacks from outside the home, and of moderate and vigorous physical activity occurred less frequently during the lockdown. Relationship satisfaction with parents, happiness, and stress from school were lower compared to before the lockdown. Smoking, stress at home, and relationship satisfaction with their best friend were equal in both cohorts. The pattern of associations within and between domains differed. More research is needed to delineate how resilient youth are on the long-term, and which youth need support to bounce back.

## Inleiding

De maatregelen om in het voorjaar van 2020 de verspreiding van het coronavirus tegen te gaan hadden grote impact op de leefsituatie van jongeren. Middelbare scholen waren gesloten, het onderwijs vond online plaats en jongeren zaten veel meer thuis. Ook hun ouders waren in veel gevallen meer thuis. Sporten in teamverband was niet mogelijk, de horeca was gesloten en feesten en festivals waren niet toegestaan. Deze situatie kan beschouwd worden als een ‘natuurlijk experiment’ waarin de sociale context aanzienlijk is veranderd. Een belangrijke vraag is of en hoe bepaalde gedragingen (eetgedrag, leefstijl) en sociaal-emotionele gezondheid van jongeren samenhangen en zijn veranderd als gevolg van een dergelijke drastische verandering in de sociale context. De focus van eerdere onderzoeken ligt veelal op sociaal-emotionele gezondheid. Ook hebben deze onderzoeken vaak de beperking dat ze slechts op één moment zijn gemeten (crosssectioneel onderzoek) en/of alleen tijdens de coronaperiode zijn uitgevoerd. Andere onderzoeken zijn wel zowel vóór als tijdens de coronacrisis uitgevoerd bij dezelfde jongeren (longitudinaal onderzoek). Het kan hierbij lastiger zijn om de ‘normale’ ontwikkeling (met het toenemen van de leeftijd) te onderscheiden van de impact van de coronamaatregelen. Ons onderzoek heeft een uniek ontwerp waarin derdeklassers van de middelbare school *voor *de coronacrisis (voorjaar van 2019) worden vergeleken met een qua demografische karakteristieken vergelijkbare groep derdeklassers *tijdens* de eerste lockdown van de coronacrisis (voorjaar 2020). We vergelijken in dit onderzoek twee cohorten op enkele domeinen die een belangrijke rol spelen in het leven van jongeren (eetgedrag, leefstijl en sociaal-emotionele gezondheid) en kijken naar de samenhang binnen en tussen de domeinen per cohort. Dit levert een belangrijke bijdrage aan eerder onderzoek, omdat het naar een breed scala van (onderlinge samenhang in) eetgedrag, leefstijl en emotionele gezondheid bij jongeren kijkt.

### Eetgedrag

Al tijdens de eerste lockdown van de coronacrisis werd er in de media gesproken over ‘coronakilo’s’, waarbij gesuggereerd werd dat jongeren en volwassenen in gewicht aankwamen. Dit kan veroorzaakt worden door het eten van meer ongezonde voeding, maar ook door minder te bewegen. Onderzoek van het Voedingscentrum liet echter zien dat 83 % van de volwassenen aangaf tijdens de eerste lockdown niet anders te zijn gaan eten. Daarnaast gaf 10 % aan gezonder te zijn gaan eten en ging de overige 7 % ongezonder eten [1]. Of dit ook geldt voor jongeren is nog niet helemaal duidelijk. De literatuur is tegenstrijdig, al lijkt er een vermindering van gezond en een toename van ongezond eetgedrag te zijn [2, 3]. Ons onderzoek levert een bijdrage aan de huidige literatuur door inname van gezonde (groente en fruit) en ongezonde (energierijke snacks en suikerhoudende dranken) producten apart in kaart te brengen voor producten verkregen vanuit de thuissituatie versus producten die buitenshuis waren verkregen (zoals op school of bij anderen thuis). Op basis van eerder gepubliceerde internationale literatuur valt te verwachten dat de consumptie van suikerhoudende dranken en ongezonde snacks – vooral thuis verkregen – hoger is in het coronacohort, dan in het cohort een jaar eerder. Hoewel jongeren meer thuis bij hun ouders zijn, passen ouders soms te strenge maatregelen toe, die vaak ineffectief blijken [3]. We verwachten dat groente- en fruitconsumptie (van thuis) tijdens de lockdown hoger is, omdat ouders mogelijk meer zicht en invloed hebben (via bijvoorbeeld hun eigen eetgedrag thuis) op wat jongeren aan fruit en groenten eten [3, 4]. De verwachting is dat gezond én ongezond snacken buitenshuis lager is door de beperkende coronamaatregelen om buitenshuis dingen te ondernemen.

### Leefstijl

Naast eetgedrag lijkt de coronacrisis ook impact gehad te hebben op de leefstijl van jongeren. Uit eerder onderzoek bleek bijvoorbeeld dat het alcoholgebruik tijdens de lockdown afgenomen was onder volwassenen en jongvolwassenen, zoals studenten [5]. Dit is waarschijnlijk te verklaren doordat de horeca gesloten was en er nauwelijks sociale activiteiten plaatsvonden waarbij normaliter alcohol wordt gedronken. Het percentage (volwassen) rokers lijkt stabiel te zijn gebleven, waarbij sommige rokers meer gingen roken (door stress of verveling) en andere juist minder rookten (vanwege gezondheidsredenen) [6]. Er is op dit moment nog weinig onderzoek gedaan naar alcohol en roken dat zich specifiek op middelbare scholieren heeft gericht, maar de verwachting is dat het alcoholgebruik tijdens de lockdown lager is, en dat er geen verschil is voor rookgedrag. Daarnaast verwachten we, in lijn met eerder onderzoek onder kinderen, adolescenten, jongvolwassenen (studenten) en volwassenen [5, 7], dat de jongeren tijdens de lockdown minder vaak en lang sportten dan de jongeren in het cohort van een jaar eerder, toen er geen beperkende coronamaatregelen waren.

### Sociaal-emotionele gezondheid

De eerste lockdown lijkt een negatieve invloed te hebben gehad op de mentale gezondheid en kwaliteit van leven van jongeren [8, 9]. Zo bleek uit longitudinale onderzoeken dat de mate van eenzaamheid tijdens de coronacrisis ten opzichte van eerdere jaren toenam [10], voornamelijk bij jongeren die onderwijs op afstand volgden [11]. Ook bleek er tijdens de lockdown sprake van meer stress, een lager geluksgevoel en een slechtere relatiekwaliteit [10–13]. Jongeren waren tijdens de eerste lockdown meer thuis met hun ouders en hadden minder (offline) contact met leeftijdgenoten, wat van invloed kan zijn geweest op de kwaliteit van de relatie met ouders en leeftijdgenoten. De effecten kunnen zowel positief (door bijvoorbeeld intensivering van de goede relatie met ouders, minder stress door school of het beperken van problematisch contact met klasgenoten), als negatief zijn (door bijvoorbeeld stress thuis, een slechte relatie met ouders of veranderende relaties met vrienden). Vooral dat laatste lijkt het geval te zijn: er werden meer conflicten met ouders gerapporteerd [11], er was minder steun van ouders ontvangen [14] en ouders rapporteerden een lagere mate van *positive parenting* [14]. De verwachting is dat de sociaal-emotionele gezondheid (onder andere geluk, stress en eenzaamheid) van jongeren tijdens de eerste lockdown minder goed is dan van een vergelijkbaar cohort een jaar eerder [10–13].

### Samenhang binnen en tussen eetgedrag, leefstijl en sociaal-emotionele gezondheid

Ten slotte is het belangrijk om inzicht te krijgen in de samenhang binnen en tussen eetgedrag, leefstijl en sociaal-emotionele gezondheidsfactoren voor en tijdens de lockdown. Eetgedrag en leefstijl lijken in belangrijke mate het welbevinden van jongeren te kunnen bepalen. Zo hadden jongeren die meer fysieke activiteit vertoonden bijvoorbeeld minder depressieve symptomen over tijd voor de coronacrisis [15] en lijkt activiteit ook tijdens de coronacrisis belangrijk te zijn voor het welbevinden van jongeren en volwassenen [16]. Tot nu toe is coronaonderzoek bij jongeren veelal gericht op welbevinden en soms op eetgedrag of leefstijl, en is er weinig coronaonderzoek dat aspecten van eetgedrag, leefstijl en welbevinden onder jongeren combineert. Dit onderzoek levert een belangrijke bijdrage omdat het inzicht geeft in de samenhang tussen constructen van eetgedrag, leefstijl en sociaal-emotionele gezondheid binnen vergelijkbare cohorten vóór en tijdens de coronacrisis. Bovendien suggereren diverse theoretische raamwerken dat de sociaal-emotionele gezondheid in grote mate wordt bepaald door de psychische behoeften van sociale verbondenheid, autonomie en zelfacceptatie [17, 18]. In lijn hiermee heeft coronaonderzoek aangetoond dat jongeren die tijdens de coronacrisis kwalitatief goede contacten hadden met zowel leeftijdgenoten als ouders een betere mentale gezondheid over tijd rapporteerden dan jongeren die dit niet hadden [9, 10, 13, 19, 20]. Dit onderstreept het belang van kwalitatief goede sociale relaties voor het mentaal welbevinden (onder andere geluk, stress, eenzaamheid). Omdat jongeren meer thuis waren en op hun ouders waren aangewezen, zou de kwaliteit van de relatie met ouders zelfs sterker kunnen samenhangen met sociaal-emotionele gezondheid. Door de kenmerkende cohortvergelijking kan ons onderzoek ook hierin eerste inzichten verschaffen.

### Het huidige onderzoek

Samenvattend richt dit onderzoek zich op de domeinen eetgedrag, leefstijl en sociaal-emotionele gezondheid en de onderlinge samenhang, waarbij een cohort jongeren dat tijdens de eerste lockdown in de derde klas zat wordt vergeleken met een cohort jongeren dat vóór de coronacrisis in de derde klas zat. Dit heeft als voordeel dat we de impact van de coronamaatregelen kunnen onderzoeken zonder dat deze effecten vertekend zijn door de voortgaande fysieke en sociaal-emotionele ontwikkeling van de jongeren. Het huidige onderzoek vult belangrijke lacunes binnen de huidige coronaliteratuur, specifiek voor de impact van de coronacrisis op jongeren in Nederland.

## Methode

### Onderzoeksopzet

Dit onderzoek maakt deel uit van het longitudinale onderzoeksproject ‘G(V)OED voor elkaar!’, waarin met verschillende vragenlijstmetingen data zijn verzameld over gezondheidsgedrag en de sociaal-emotionele gezondheid van jongeren en hun ouders [4]. Tijdens de eerste meting (meetmoment T1: najaar 2017) zijn zowel eersteklassers (cohort 1) als tweedeklassers (cohort 2) van het voortgezet onderwijs geïncludeerd, die longitudinaal zijn gevolgd. In dit onderzoek worden de twee cohorten vergeleken op het moment dat ze in het derde leerjaar zaten. Cohort 1 zat in leerjaar 3 tijdens de eerste lockdown van de coronacrisis (meetmoment T4) en wordt vergeleken met cohort 2, dat een jaar eerder in leerjaar 3 zat (meetmoment T3).

### Participanten en procedure

Jongeren zijn geworven via zeven middelbare scholen in het zuiden en oosten van Nederland. Ouders ontvingen een informatiebrief en werden gevraagd om toestemming voor deelname te verlenen voor zowel hun kind als henzelf. Ook gaven jongeren zelf toestemming. In totaal werd op T1 voor 718 jongeren toestemming verkregen. Data van ouders zijn in het huidige onderzoek buiten beschouwing gelaten.

De dataverzameling vond plaats met Qualtrics, een online softwareapplicatie om vragenlijsten via computers af te nemen. Op T3 (voorjaar 2019) werd de dataverzameling in de desbetreffende klassen op scholen uitgevoerd. Op T4 (najaar 2020) werden de adolescenten gevraagd om de vragenlijsten op een zelfgekozen moment online in te vullen. Op T4 werd gevraagd om retrospectief te rapporteren over de situatie tijdens de eerste lockdown in voorjaar 2020. Als waardering voor deelname werden onder de deelnemers cadeaus verloot (variërend in waarde van 10 tot 250 euro).

Het onderzoek is onafhankelijk getoetst door de Ethiekcommissie Sociale Wetenschappen (ECSW20170805-516) van de Radboud Universiteit. Er is formeel geen bezwaar tegen dit onderzoek. Bij aanvang van het onderzoek werd iedereen duidelijk gemaakt dat deelname vrijwillig was, dat er op ieder moment gestopt kon worden en dat alle data gepseudonimiseerd verwerkt zouden worden.

In cohort 1 (*N* = 177) was de verhouding leerlingen van het vmbo (28,8 %) en havo/vwo (71,2 %) anders dan in cohort 2 (*N* = 237): vmbo (43,5 %) en havo/vwo (56,5 %). Omdat opleidingsniveau kan samenhangen met de variabelen die we onderzochten [21], hebben we de verhouding gelijk gemaakt door in het grootste cohort (cohort 2) 54 deelnemers random te selecteren uit de 103 vmbo-leerlingen, waarmee de verhouding vmbo versus havo/vwo ook 28,7 % versus 71,3 % is geworden (*N* = 188).

### Instrumenten

Op T3 en T4 werden dezelfde vragenlijsten gebruikt, waarbij op T3 veelal de meer algemeen geldende situaties en op T4 de periode van de eerste lockdown (gedefinieerd als: ‘de periode van half maart 2020 tot half juni 2020 toen scholen gesloten waren en er alleen online onderwijs was vanwege het coronavirus’) werden uitgevraagd. De vragenlijst richtte zich op de volgende domeinen: eet- en drinkgedrag (suikerhoudende non-alcoholische dranken, gezonde en ongezonde snacks), leefstijl (roken, alcohol, fysieke activiteit), sociaal-emotionele gezondheid (eenzaamheid, relatietevredenheid ouders en beste vriend(in), geluk). De vraagstelling en codering wordt beschreven in de aanvullende digitale content Supplement Methode.

### Analyses

Eerst zijn van beide cohorten voor alle variabelen beschrijvende statistieken berekend. Het betreft gemiddelden en standaarddeviaties voor continue variabelen en percentages voor ordinale of dichotome variabelen. Om de twee cohorten in het voorjaar van 2019 (T3) en voorjaar van 2020 (T4) met elkaar te vergelijken zijn vervolgens onafhankelijke *t*-toetsen uitgevoerd voor de continue variabelen en chi-kwadraattoetsen voor de ordinale of dichotome variabelen. Om rekening te houden met en te corrigeren voor het aantal tests dat werd uitgevoerd, is op de *p*-waarden een Bonferroni-correctie toegepast. Afhankelijk van het aantal tests dat is uitgevoerd, werden er verschillende significantieniveaus gehanteerd; een significantieniveau van *p* < 0,008 (0,05/6) voor drinkgedrag, *p* < 0,004 (0,05/14) voor eetgedrag, *p* < 0,008 (0,05/6) voor leefstijl en *p* < 0,008 (0,05/6) voor sociaal-emotionele gezondheid. Ten slotte werd de samenhang tussen de variabelen onderling berekend. Standaard werd de Pearson-correlatiecoëfficiënt gebruikt, maar bij ordinale of twee dichotome variabelen werden Spearman-correlaties gehanteerd. Bij de interpretatie van de correlaties werd een Bonferroni-gecorrigeerde *p*-waarde van *p* < 0,001 (0,05/35) gehanteerd, rekening houdend met het aantal variabelen. Bij alle analyses werd een *p*-waarde tussen 0,05 en de Bonferroni-gecorrigeerde *p*-waarde als ‘niet significant na correctie’ beschouwd, en een *p*-waarde kleiner dan de Bonferroni-gecorrigeerde *p*-waarde als significant. Alle analyses zijn uitgevoerd met SPSS (versie 25).

## Resultaten

### Vergelijking van cohorten vóór en tijdens de eerste lockdown

Tabel 1 laat zien dat beide cohorten vergelijkbaar zijn op demografische gegevens. De resultaten voor drinkgedrag (tab. 2) tonen een consistent patroon, waarbij de consumptie van suikerhoudende dranken van buitenshuis tijdens de eerste lockdown lager was dan ervoor, terwijl de consumptie van thuis niet verschilde. Wat betreft eetgedrag (tab. 3) zien we een vergelijkbaar patroon voor ongezond snackgedrag van buitenshuis, dat consequent lager lag tijdens de eerste lockdown. De consumptie van zoete snacks (gebak, cake en grote koeken, chocolade) van thuis lijkt daarentegen hoger te zijn tijdens de eerste lockdown (niet-significant na correctie). De fruitinname ligt tijdens de eerste lockdown lager (vergeleken met voor de coronacrisis), terwijl er geen verschillen werden gevonden voor de consumptie van warme en rauwe groenten. Verder blijkt dat jongeren minder matige en zware fysieke activiteit rapporteerden tijdens de eerste lockdown (tab. 4). Daarnaast was het percentage jongeren dat wekelijks alcohol drinkt hoger (dit was na correctie echter niet significant). Er werd geen verschil gevonden in het (zeer lage) percentage rokers vóór de coronacrisis en tijdens de eerste lockdown. Ten slotte blijkt dat jongeren meer gevoelens van eenzaamheid (na correctie niet significant), minder relatietevredenheid met ouders, minder stress door school en een lagere mate van geluk rapporteerden tijdens de eerste lockdown, dan vóór de coronacrisis (tab. 5). Er werden geen verschillen gevonden voor de relatietevredenheid met de beste vriend(in) en de ervaren stress in de thuissituatie.

**Table 1:** 

	voorjaar 2019	voorjaar 2020	*t *of χ^2^	*p*
geslacht (% meisje)	51,40 %	55,90 %	0,72	0,395
leeftijd	15,00 (0,40)	14,95 (0,45)	1,14	0,256
opleidingsniveau (% vmbo)	28,80 %	28,70 %	<0,01	0,985

**Table 2:** 

	voorjaar 2019	voorjaar 2020	*t*	*p*
frisdrank met suiker				
– van thuis	3,05 (2,16)	2,95 (2,48)	0,38	0,701
– van buitenshuis	2,27 (2,59)	0,98 (1,75)	5,50	<0,001*
energiedrankjes				
– van thuis	0,10 (0,48)	0,10 (0,61)	0,10	0,924
– van buitenshuis	0,61 (1,70)	0,26 (1,11)	2,34	**0,020**
zoete melkdrankjes				
– van thuis	1,65 (2,25)	1,54 (2,26)	0,46	0,643
– van buitenshuis	0,58 (1,59)	0,14 (0,50)	3,45	0,001*

Significante veranderingen bij hantering *p* < 0,05 zijn vetgedrukt, significante veranderingen bij hantering *p* < 0,008 (Bonferroni-gecorrigeerd) worden met een asterisk aangeduid

**Table 3:** 

	voorjaar 2019	voorjaar 2020	*t*	*p*
gebak, cake of grote koeken				
– van thuis	2,44 (2,05)	3,00 (2,16)	2,56	** 0,011**
– van buitenshuis	1,49 (1,81)	0,72 (1,42)	4,43	<0,001*
candybars				
– van thuis	1,24 (1,63)	1,17 (1,79)	0,40	0,691
– van buitenshuis	0,94 (1,86)	0,41 (1,28)	3,16	0,002*
chocolade				
– van thuis	1,77 (1,68)	2,17 (2,03)	2,07	** 0,039**
– van buitenshuis	1,13 (2,02)	0,53 (1,43)	3,23	0,001*
warme snacks				
– van thuis	1,13 (0,85)	1,19 (1,05)	0,57	0,568
– van buitenshuis	0,93 (1,25)	0,49 (1,08)	3,65	<0,001*
fruit				
– van thuis	4,97 (2,17)	4,48 (2,34)	2,06	** 0,040**
– van buitenshuis	0,79 (1,73)	0,29 (0,72)	3,52	0,001*
rauwe groenten				
– van thuis	3,76 (2,24)	3,89 (2,27)	0,53	0,600
– van buitenshuis	0,50 (1,49)	0,31 (0,96)	1,44	0,150
warme groenten				
– van thuis	5,03 (1,69)	4,81 (1,96)	1,13	0,258
– van buitenshuis	0,51 (1,60)	0,28 (1,01)	1,61	0,109

Significante veranderingen bij hantering *p* < 0,05 zijn vetgedrukt, significante veranderingen bij hantering *p* < 0,004 (Bonferroni-gecorrigeerd) worden met een asterisk aangeduid

**Table 4:** 

	voorjaar 2019	voorjaar 2020	*t *of χ^2^	*p*
alcohol (% wekelijks)	4,00 %	9,20 %	3,99	** 0,046**
roken (% wekelijks)	2,80 %	1,80 %	0,43	0,511
zware fysieke inspanning				
– dagen per week	3,28 (1,69)	1,92 (1,86)	7,22	<0,001*
– tijd per keer in categorieën	4,24 (1,69)	2,77 (1,50)	8,29	<0,001*
matige fysieke inspanning				
– dagen per week	4,68 (1,97)	2,49 (2,16)	10,36	<0,001*
– tijd per keer in categorieën	3,68 (1,59)	2,63 (1,49)	6,01	<0,001*

Significante veranderingen bij hantering *p* < 0,05 zijn vetgedrukt, significante veranderingen bij hantering *p* < 0,008 (Bonferroni-gecorrigeerd) worden met een asterisk aangeduid

**Table 5:** 

	voorjaar 2019	voorjaar 2020	*t*	*p*
eenzaamheid	1,38 (0,50)	1,51 (0,52)	2,51	** 0,013**
stress				
– thuis	1,90 (0,76)	1,76 (0,79)	1,68	0,078
– door school	2,46 (0,81)	2,04 (0,90)	4,59	<0,001*
relatietevredenheid				
– met ouders	91,81 (13,03)	86,72 (17,54)	2,98	0,003*
– met beste vriend(in)	90,18 (12,98)	91,09 (13,89)	0,63	0,526
geluk	7,84 (1,24)	7,23 (1,66)	3,93	<0,001*

Significante veranderingen bij hantering *p* < 0,05 zijn vetgedrukt, significante veranderingen bij hantering *p* < 0,008 (Bonferroni-gecorrigeerd) worden met een asterisk aangeduid

### Samenhang tussen variabelen binnen en tussen de domeinen

**Figure 1:**
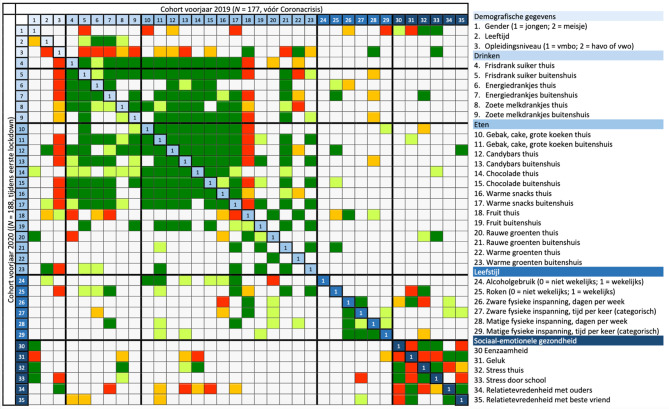

Om in kaart te brengen of de variabelen onderling zowel binnen de domeinen (eetgedrag, leefstijl en sociaal-emotionele gezondheid), als tussen de domeinen samenhangen, zijn correlatiecoëfficiënten berekend binnen het cohort vóór de coronacrisis (in fig. 1 en in digitaal aanvullende content tab. S1 boven de diagonaal) en binnen het cohort tijdens de eerste lockdown (in fig. 1 en in digitaal aanvullende content tab. S1 onder de diagonaal).

#### Eetgedrag (variabelen 4 tot en met 23 in fig. 1 en de digitaal aanvullende content tab. S1)

De variabelen die de inname van suikerhoudende dranken (variabelen 4 tot en met 9) en ongezonde snacks (variabelen 10 tot en met 17) reflecteren hangen sterk positief samen, hoewel die samenhang tijdens de eerste lockdown minder consistent en sterk lijkt te zijn dan een jaar eerder. Wat verder opvalt is dat de consumptie van ongezonde snacks vóór de coronacrisis negatief samenhing met de inname van fruit (kolom 18), maar dat dit niet het geval bleek tijdens de eerste lockdown (rij 18).

#### Leefstijl (variabelen 24 tot en met 29 in fig. 1 en de digitaal aanvullende content tab. S1)

De correlatiecoëfficiënten laten zien dat tijdens de eerste lockdown alle maten van fysieke activiteit (variabelen 26 tot en met 29) met elkaar samenhingen, terwijl vóór de coronacrisis alleen aantal dagen per week en tijd per keer van zware inspanning samenhingen.

#### Sociaal-emotionele gezondheid (variabelen 30 tot en met 35 in fig. 1 en de digitaal aanvullende content tab. S1)

Binnen het domein sociaal-emotionele gezondheid lieten de onderlinge relaties in beide cohorten grotendeels hetzelfde patroon zien. Wel was het zo dat de relatietevredenheid met ouders (variabele 34) tijdens de eerste lockdown samenhing met minder gevoelens van eenzaamheid (variabele 30), terwijl dit verband vóór de coronacrisis niet werd gevonden. De relatietevredenheid met de beste vriend(in) (variabele 35) hing daarnaast alleen vóór de coronacrisis, en niet tijdens de eerste lockdown, samen met minder gevoelens van stress thuis en door school (variabelen 32 en 33).

#### Samenhang over thema’s heen (gehele fig. 1 en de digitaal aanvullende content tab. S1)

Meisjes (variabele 1) rapporteerden zowel vóór als tijdens de eerste lockdown gemiddeld een minder goede sociaal-emotionele gezondheid (variabelen 30 tot en met 33) in vergelijking met jongens. Een hoger opleidingsniveau (variabele 3) hing samen met het consumeren van minder suikerhoudende dranken (variabelen 4 tot en met 9) en ongezonde snacks (variabelen 10 tot en met 17). Dit verband lijkt sterker tijdens de eerste lockdown vergeleken met een jaar eerder vóór de coronacrisis. Ook hing opleidingsniveau samen met ervaren stress door school (variabele 33). Vóór de coronacrisis ervaarden jongeren met een hoger opleidingsniveau meer stress door school, terwijl juist de jongeren met een lager opleidingsniveau tijdens de eerste lockdown meer stress door school ervaarden.

Voor de samenhang tussen constructen van eetgedrag en sociaal-emotionele gezondheid valt op dat nauwelijks verbanden werden gevonden vóór de coronacrisis. Dit was anders tijdens de eerste lockdown, waar de consumptie van bepaalde suikerhoudende dranken en ongezonde snacks (variabelen 7, 8, 13, 14, en 15) samenhing met meer stress thuis (variabele 32) en minder relatietevredenheid met ouders (variabele 34).

Als jongeren op meer dagen per week fysiek actief waren (variabele 26) rapporteerden ze voor de coronacrisis meer gevoelens van geluk (variabele 31) en minder stress thuis (variabele 32), maar niet tijdens de eerste lockdown. Daarnaast valt op dat het wekelijkse alcoholgebruik (variabele 24) tijdens de eerste lockdown positief samenhangt met de inname van verschillende ongezonde snacks (variabelen 10 tot en met 17), terwijl deze verbanden niet zichtbaar waren vóór de coronacrisis.

## Beschouwing

Op basis van het unieke onderzoeksontwerp, waarin derdeklassers van de middelbare school tijdens de eerste lockdown van de coronacrisis werden vergeleken met een vergelijkbare groep derdeklassers vóór de coronacrisis, kunnen we concluderen dat sommige gedragingen tijdens de eerste lockdown frequenter voorkwamen (de consumptie van zoete snacks thuis, wekelijks alcoholgebruik) en dat er in die periode meer gevoelens van eenzaamheid waren. Andere gedragingen kwamen juist minder vaak voor tijdens de eerste lockdown (de consumptie van suikerhoudende dranken en ongezonde snacks buitenshuis, fruit thuis, matige en zware fysieke activiteit). De relatietevredenheid met ouders en geluksgevoel waren lager, evenals stress door school. Roken, stress in de thuissituatie en de relatietevredenheid met de beste vriend(in) waren in beide cohorten even hoog. Het patroon van samenhang binnen en tussen de constructen leverde een aantal interessante inzichten op die hieronder wordt besproken.

Het eetpatroon van jongeren lijkt tijdens de lockdown in de thuissituatie ongezonder te zijn dan daarvoor. In tegenstelling tot de hypotheses en eerdere literatuur wordt er minder fruit geconsumeerd, hetgeen kan komen door een gebrek aan structuur (bijvoorbeeld het meebrengen van fruit van thuis voor tijdens de schoolpauze) [3]. In overeenstemming met de hypotheses wordt er meer ongezond gesnackt, mogelijk door verveling [22], meer stress van ouders thuis [23] en de constante beschikbaarheid van snacks thuis, waardoor automatische, ongezonde patronen zouden kunnen worden getriggerd [24]. De consumptie van snacks en zoete drankjes die buitenshuis worden verkregen, is drastisch afgenomen, wat logisch te verklaren is uit het feit dat jongeren door de coronamaatregelen meer thuis waren. Het valt ook op dat de consumptie van ongezonde snacks en suikerhoudende drankjes tijdens de eerste lockdown samenhing met minder relatietevredenheid met ouders, minder gevoelens van geluk en meer stress thuis, terwijl dit een jaar eerder (vóór de coronacrisis) niet het geval was. Eerder onderzoek wijst uit dat ouderlijke stress een bron kan zijn voor ongezonder eetgedrag bij jongeren [23]. We dienen echter voorzichtig te zijn met de interpretatie van verschillen in samenhang tussen cohorten, omdat dit niet is getoetst.

Wat betreft leefstijl lijkt wekelijks alcoholgebruik tijdens de lockdown hoger te zijn dan vóór de coronacrisis, hoewel dit na correctie niet significant was. Dit is niet in lijn met onze hypotheses en de literatuur, waarin een afname in alcoholgebruik in Nederlandse jongvolwassenen tijdens de coronacrisis werd gerapporteerd [5]. Internationale onderzoeken tonen tegenstrijdige resultaten onder jongeren – er was bijvoorbeeld een afname in alcoholgebruik in Brazilië [25] en geen verandering in de Verenigde Staten [26]. Opvallend is dat alcoholgebruik tijdens de coronacrisis positief samenhing met ongezond eetgedrag, terwijl dit in het vergelijkbare eerdere cohort niet het geval was. Het is mogelijk dat een eenzijdige (thuis)context zorgt voor een clustering van ongezonde gedragingen. Dat jongeren tijdens de coronacrisis minder fysiek actief zijn is in lijn met de hypothese en andere onderzoeken [5, 7, 27], en kan verklaard worden door de restricties wat betreft activiteiten binnen sportclubs. Voorts valt op dat de frequentie van fysieke activiteit vóór de coronacrisis positief samenhing met welbevinden (meer geluksgevoelens en minder stress thuis), terwijl dit tijdens de lockdown niet het geval was. Het is bekend dat fysieke activiteit gevoelens van welbevinden kan stimuleren [15], maar omdat jongeren tijdens de coronacrisis minder sportten is de hoeveelheid fysieke activiteit mogelijk onvoldoende geweest om deze gevoelens te stimuleren of werden ze overschaduwd door de negatieve impact van de coronamaatregelen, de verminderde relatietevredenheid met ouders en/of angst voor het coronavirus.

Jongeren geven hun leven tijdens de lockdown gemiddeld een lager gelukscijfer dan een vergelijkbaar cohort een jaar eerder. Dit is waarschijnlijk te wijten aan alle beperkende coronamaatregelen tijdens de lockdown en komt overeen met een onderzoek dat in drie verschillende populaties een lager geluksgevoel beschreef [12]. De relatietevredenheid met ouders was tijdens de lockdown lager en hing samen met meer eenzaamheid in deze periode, maar niet daarvoor. Een verklaring hiervoor kan gevonden worden in een onderzoek onder jongeren waarbij zowel ouders als de jongeren zelf rapporteerden dat de sfeer thuis tijdens de lockdown slechter was dan daarvoor en dat er meer negatieve ouder-kindinteracties plaatsvonden [14, 28]. Verder rapporteerden jongeren tijdens de lockdown minder stress door school. Dit kan met het (online) onderwijs zelf te maken hebben, maar ook met de afwezigheid van andere vormen van stress door school, zoals pesten en problemen in de omgang met leeftijdgenoten. Zo werd inderdaad aangetoond dat jongeren zich minder gepest voelden [11]. Opvallend is dat er in het cohort voor de coronacrisis een positieve samenhang was tussen opleidingsniveau en stress door school (dus hoger opleidingsniveau, meer schoolstress), terwijl dit tijdens de lockdown andersom was (lager opleidingsniveau, meer schoolstress). Dit suggereert dat jongeren op het vmbo meer stress ervaarden tijdens de schoolsluiting en het afstandsonderwijs dan jongeren op de havo/het vwo. Voor toekomstige preventie- en interventieprogramma’s is het belangrijk om te achterhalen wat die bron van stress door school is, en hierbij onderscheid te maken tussen opleidingsniveaus.

### Sterke punten en beperkingen

De sterke punten van dit onderzoek zijn dat ons onderzoek een uniek onderzoeksontwerp heeft waarin derdeklassers van de middelbare school vóór de coronacrisis (voorjaar 2019) werden vergeleken met een wat betreft demografische karakteristieken vergelijkbare groep derdeklassers tijdens de eerste lockdown van de coronacrisis (voorjaar 2020). Hiermee controleren we voor de eventuele ontwikkelingsspecifieke effecten door de voortgaande sociaal-emotionele ontwikkeling van de jongeren. Bovendien hebben wij in ons onderzoek veel verschillende relevante variabelen binnen de belangrijke dimensies van gezondheid (fysieke, mentale en sociale gezondheid) in kaart gebracht.

Natuurlijk kent ons onderzoek ook beperkingen. Ten eerste worden naast de onderzochte factoren ook internetgebruik en schermtijd gezien als belangrijke factoren die een rol kunnen spelen in het welzijn van jongeren (zie de website van het Trimbos-instituut: https://www.trimbos.nl/kennis/zijn-jongeren-gelukkig), maar deze data waren in ons onderzoek niet beschikbaar. Verder wordt er in het onderzoek gebruikgemaakt van zelfrapportage. Zelfrapportage kan onderhevig zijn aan beperkingen, zoals sociaal-wenselijke antwoorden, beperkt introspectief vermogen van de deelnemer en uiteenlopende interpretaties van de vragen. Daarnaast is het niet mogelijk uitspraken te doen over de causaliteit van de eerste lockdown op de gevonden cohortverschillen in het huidige onderzoek. Ook is het een beperking dat de situatie tijdens de eerste lockdown (voorjaar 2020) retrospectief (najaar 2020) is uitgevraagd. Deze rapportage vond echter plaats tijdens de coronacrisis. Daardoor is deze minder onderhavig aan zogenoemde contextafhankelijke vertekende geheugenprocessen, die vaak het probleem vormen bij gebruik van retrospectieve rapportages [29, 30]. Ten slotte heeft dit onderzoek alleen gekeken naar de situatie tijdens de eerste lockdown (vergeleken met een cohort van een jaar daarvoor) bij Nederlandse jongeren. De resultaten kunnen dus niet worden gegeneraliseerd naar jongeren in andere landen en naar andere periodes tijdens de coronacrisis, waarin mogelijk andere maatregelen en restricties golden.

## Conclusie

De maatregelen om in het voorjaar van 2020 de verspreiding van het coronavirus tegen te gaan door de eerste lockdown hadden grote impact op de leefstijl en sociale mogelijkheden die het welbevinden van jongeren beïnvloeden. Over het algemeen lijken jongeren er tijdens de coronacrisis minder goed voor te staan dan een vergelijkbaar cohort een jaar eerder (meer ongezond snackgedrag, minder sporten, minder goed mentaal welbevinden). Veel lijkt te verklaren door de ingrijpende coronamaatregelen en de verandering van context. Aan de andere kant waren roken, stress thuis en relatietevredenheid met de beste vriend(in) in beide cohorten even hoog. Verder onderzoek dient vooral uit te wijzen hoe veerkrachtig jongeren op de langere termijn zijn en welke (subgroepen) jongeren ondersteuning nodig hebben om terug te veren. Voor de praktijk is het belangrijk te weten welke domeinen tijdens de lockdown bij jongeren verslechterden, verbeterden of stabiel bleven. Hierbij kan de lockdown beschouwd worden als een grote maatschappelijke stressor, die ook in de toekomst in gelijke of andere vorm kan voorkomen. Inzicht in veranderende en gelijkblijvende processen biedt houvast aan de praktijk om risico- en beschermende factoren in jongeren en hun gezinnen te identificeren en hierop in te grijpen om het welzijn van jongeren te maximaliseren.
