# Supplementary material for: A cohort-comparison of food intake, lifestyle and social-emotional health of adolescents before and during the corona crisis
Source: TSG. 2022 Mar 8;100(2):40–8. [Article in Dutch] doi: 10.1007/s12508-022-00331-4 (PMC8902735; doi:10.1007/s12508-022-00331-4)
Supplement: Supplementary file 1 [file 12508_2022_331_MOESM1_ESM.doc]

**Supplement Methode behorend bij ‘Een Cohortvergelijking van Eetgedrag, Leefstijl en Sociaal-Emotionele Gezondheid bij Jongeren vóór en tijdens de Coronacrisis’**

**Eetgedrag en Drinkgedrag**

Om eet- en drinkgedrag te meten werd gebruik gemaakt van een voedselfrequentievragenlijst (FFQ, zie [1]). Tijdens het invullen van de FFQ werd gevraagd naar de inname van (1) frisdranken met suiker (met en zonder koolzuur, bijv. cola of limonade; lightproducten uitgesloten); (2) energiedrankjes (bijv. Energy of Red Bull); (3) zoete melkdrankjes (bijv. Fristi of Yogho Yogho; lightproducten uitgesloten); (4) gebak, cake en/of grote koeken (bijv. donut of muffin); (5) candybars (bijv. Nuts of Mars); (6) chocolade (bijv. reep chocolade of bonbons); (7) warme en/of gefrituurde snacks (bijv. worstenbroodje of stuk pizza); (8) fruit (bijv. appel of banaan); en (9) rauwe groenten (bijv. sla of snoeptomaatjes); en (10) warme groenten (gekookt, gebakken, gestoomd of anderszins verwarmd; bijv. broccoli of sperziebonen). Voor elk item werd jongeren gevraagd aan te geven hoeveel dagen per week (0 – 7 dagen) ze het desbetreffende product in vier verschillende contexten verkregen: (1) van huis gepakt of gekregen; (2) op school gekocht; (3) ergens anders gekocht; en (4) ergens anders gekregen. Naast het eet- en drinkgedrag “van thuis” (context 1), werd het eet- en drinkgedrag “van buitenshuis” gedefinieerd als de som van de scores op context 2, 3, en 4. Hoe hoger de score, hoe frequenter jongeren de desbetreffende producten consumeren.

**Leefstijl**

*Alcoholgebruik.* Aan de jongeren werd gevraagd welke stelling het meest van toepassing was: (1) ik heb nog nooit alcohol gedronken, niet eens een slokje; (2) ik heb een paar keer een slokje alcohol gedronken; (3) ik heb vroeger (regelmatig) alcohol gedronken, maar ik drink nu niet meer; (4) ik drink één keer per week alcohol of minder; (5) ik drink meerdere keren per week alcohol; (6) ik drink dagelijks alcohol [2]. Op basis van deze antwoorden zijn jongeren ingedeeld in regelmatig drinken: ja (categorie 5 en 6) versus nee (categorie 1,2,3,4).

*Roken.* Aan de jongeren werd gevraagd welke stelling het meest van toepassing was: (1) ik heb nog nooit gerookt, niet eens een trekje; (2) ik heb een paar keer een trekje van een sigaret genomen; (3) ik heb vroeger (regelmatig) gerookt, maar ik ben nu gestopt met roken; (4) ik rook één keer per week of minder; (5) ik rook meerdere keren per week; (6) ik rook dagelijks [2]. Op basis van deze antwoorden zijn jongeren in gedeeld in regelmatig roken: ja (categorie 5 en 6) versus nee (categorie 1,2,3,4).

*Fysieke inspanning.* Op T3 werd lichamelijke activiteit gemeten door te vragen hoeveel dagen per week en hoeveel tijd per keer (in uren en/of minuten) ze zware inspanningen of matige inspanningen verrichten. De toelichting bij zware inspanning was dat het zorgt voor een veel snellere ademhaling, zoals hockey, wielrennen of tennis. De toelichting bij matige inspanning was dat het zorgt voor een iets snellere ademhaling, zoals fietsen of dubbeltennis. De vragen en toelichting zijn overeenkomstig met de gevalideerde “International Physical Activity Questionnaire” (korte versie) [3]. Op T4 werden dezelfde vragen gesteld, en konden jongeren ook aangeven hoeveel dagen per week ze deze inspanningen verrichten, alleen werd de tijd per keer uitgevraagd in de categorieën: 0 uur, 0,5 uur, 1 uur, 1,5 uur, 2 uur, 2,5 uur, of 3 uur of meer. De gerapporteerde tijden op T3 zijn ingedeeld in deze zelfde categorieën.

**Sociaal-Emotionele Gezondheid**

*Eenzaamheid.* Om de mate van ervaren gevoelens van eenzaamheid te meten werd de Leuvense Eenzaamheidsschaal voor Kinderen en Adolescenten gebruikt [4]. Deze schaal bestaat uit 12 items waarbij op een 4-puntslikertschaal (1 = ‘nooit’, 4 = ‘vaak’) vragen beantwoord werden rondom eenzaamheid in de relatie met leeftijdsgenoten. Een voorbeeld item is “Ik vind dat ik minder vriend(inn)en heb dan anderen”. Hoe hoger de score op deze schaal, hoe meer eenzaamheid de adolescent ervaarde. De betrouwbaarheid was 0.92 op T3 en 0.88 op T4 (Cronbach’s alfa).

*Ervaren stress.* De mate van stress werd uitgevraagd middels twee items over ervaren stress thuis en op school [5,6]. Op T3 werd gerapporteerd over het afgelopen jaar (“Hoe vaak heb je in het afgelopen jaar thuis stress ervaren?”). Op T4 werd gerapporteerd over de periode tijdens de lockdown (“Hoe vaak heb je tijdens de eerste lockdown door school stress ervaren?”). Aan de hand van een 4-puntslikertschaal (1 = ‘nooit, 4 = ‘constant’) werd de frequentie van stress in kaart gebracht. Hoe hoger de score, hoe hoger de mate van ervaren stress.

*Relatietevredenheid met beste vriend(in).* De relatietevredenheid met de beste vriend(in) van dezelfde jaarlaag op school werd gemeten met het item “Hoe tevreden ben jij met je vriendschap met je beste vriend(in)?” [7,8]. Adolescenten konden op een continue horizontale schaal van 0 tot 100 hun antwoord geven, variërend van ‘helemaal niet tevreden’ (score 0) tot ‘helemaal tevreden’ (score 100). Hoe hoger de score, hoe meer tevredenheid over de relatie met de beste vriend(in).

*Relatietevredenheid met ouders.* Relatietevredenheid ten opzichte van ouders/verzorgers werd gemeten aan de hand van vragen over de relatietevredenheid met de belangrijkste volwassenen van het huis waar ze het grootste deel van hun tijd wonen. Dit werd grotendeels over de biologische ouders gerapporteerd (>96% op T3 en >92% op T4). De vraag die gesteld werd was: “Hoe tevreden ben jij over je relatie met ouder/verzorger 1 uit huis 1?” [7,8]. Adolescenten konden op een continue horizontale schaal van 0 tot 100 hun antwoord geven, variërend van ‘helemaal niet tevreden’ (score 0) tot ‘helemaal tevreden’ (score 100). Hoe hoger de score op een item, hoe meer tevreden een adolescent was over de relatie met de ouders. De Cronbach’s alfa voor betrouwbaarheid was 0.84 op zowel T3 als T4.

*Geluk.* De mate van ervaren geluk werd uitgevraagd door middel van de levenskwaliteit ladder van Cantril [9]. Bij deze vraag werd adolescenten gevraagd hun eigen leven een cijfer te geven. De schaal liep van het ‘slechtste leven dat je je kunt voorstellen’ (1) tot het ‘beste leven dat je je kan voorstellen’ (10). Een hogere score betekent een hogere ervaren levenskwaliteit.

**Literatuur Supplement Methode**

1. Van den Broek N, Larsen JK, Verhagen M et al. Is adolescents’ food intake associated with exposure to the food intake of their mothers and best friends? Nutrients. 2020;12:786.

2. Mies GW, Treur JL, Larsen JK et al. The prevalence of food addiction in a large sample of adolescents and its association with addictive substances. Appetite. 2017;118:97-105.

3. Craig CL, Marshall AL, Sjöström M et al.. International physical activity questionnaire: 12-country reliability and validity. Med Sci Sports Exerc. 2003;35:1381-1395.

4. Marcoen A, Goossens L. De Leuvense Eenzaamheidsschaal voor Kinderen en Adolescenten. Ned Tijdschr Psychol. 1990;45:288-292.

5. Willemsen G, Vink JM, Abdellaoui A et al. The adult Netherlands Twin Register: Twenty-five years of survey and biological data collection. Twin Res Hum Genet. 2013;16:271-281.

6. Mies GW, Verweij KJH, Treur JL et al. Polygenic risk for alcohol consumption and its association with alcohol-related phenotypes: Do stress and life satisfaction moderate these relationships? Drug Alcohol Depend. 2018;183:7-12.

7. Parker JG, Asher SR. Friendship and friendship quality in middle childhood: Links with peer group acceptance and feelings of Lloneliness and social dissatisfaction. Dev Psychol. 1993;29:611-621.

8. Verhagen M, Lodder GMA, Baumeister RF. Unmet belongingness needs but not high belongingness needs alone predict adverse well-being: A response surface modeling approach. J Pers. 2018;86:498-507.

9. Cantril H. The pattern of human concerns. New Brunswick, NJ: Rutgers University Press; 1965.
