# Supplementary material for: A cohort-comparison of food intake, lifestyle and social-emotional health of adolescents before and during the corona crisis
Source: TSG. 2022 Mar 8;100(2):40–8. [Article in Dutch] doi: 10.1007/s12508-022-00331-4 (PMC8902735; doi:10.1007/s12508-022-00331-4)
Supplement: Supplementary file 2 [file 12508_2022_331_MOESM2_ESM.docx]

Supplementaire Tabel S1

*Correlatiecoëfficiënten voor Studievariabelen van Jongeren uit Leerjaar 3, in Voorjaar 2019 (N = 177, vóór Coronacrisis) en Voorjaar 2020 (N = 188, tijdens Eerste Lockdown)*

|  | Cohort voorjaar 2019 (*N* = 177, vóór Coronacrisis) | | | | | | | | | | | | | | | | | | | | | | | | | | | | | | | | | | | |
| --- | --- | --- | --- | --- | --- | --- | --- | --- | --- | --- | --- | --- | --- | --- | --- | --- | --- | --- | --- | --- | --- | --- | --- | --- | --- | --- | --- | --- | --- | --- | --- | --- | --- | --- | --- | --- |
| Cohort voorjaar 2020 ((*N* = 188, tijdens eerste lockdown) |  | 1 | 2 | 3 | 4 | 5 | 6 | 7 | 8 | 9 | 10 | 11 | 12 | 13 | 14 | 15 | 16 | 17 | 18 | 19 | 20 | 21 | 22 | 23 | 24 | 25 | 26 | 27 | 28 | 29 | 30 | 31 | 32 | 33 | 34 | 35 |
|  | 1 | 1 | -.02 | .03 | -.07 | -.29 | -.02 | -.09 | -.05 | -.12 | -.25 | -.10 | -.16 | -.08 | -.05 | .02 | -.06 | -.24 | .09 | -.04 | .05 | .06 | .06 | .03 | -.09 | .03 | -.17 | -.11 | .01 | -.23 | .18 | -.27 | .22 | .26 | -.02 | -.03 |
|  | 2 | -.17 | 1 | -.07 | <.01 | .16 | .25 | .28 | .16 | .06 | -.05 | .09 | .02 | .02 | .04 | .07 | .03 | .12 | -.12 | .01 | .05 | .11 | -.08 | .04 | .11 | -.02 | -.01 | .02 | -.12 | -.13 | -.08 | .12 | .09 | .03 | -.02 | -.07 |
|  | 3 | <.01 | -.34 | 1 | -.13 | -.29 | -.26 | -.27 | -.17 | -.29 | -.07 | -.17 | -.18 | -.33 | .01 | -.16 | -.05 | -.14 | .41 | -.08 | .12 | -.20 | .20 | -.17 | .13 | .02 | .06 | -.05 | .25 | .05 | .07 | .01 | .07 | .21 | -.04 | -.14 |
|  | 4 | -.08 | -.01 | -.21 | 1 | .40 | .19 | .28 | .35 | .25 | .27 | .25 | .44 | .28 | .31 | .27 | .30 | .21 | -.30 | -.06 | -.16 | .14 | -.15 | <.01 | .05 | .08 | -.10 | -.04 | .02 | .06 | -.03 | .03 | -.06 | -.04 | .11 | .12 |
|  | 5 | -.05 | .02 | -.21 | .27 | 1 | .19 | .57 | .24 | .54 | .22 | .58 | .41 | .66 | .29 | .59 | .26 | .50 | -.28 | .09 | .04 | .40 | -.20 | .17 | .13 | .14 | .03 | .08 | -.17 | .15 | -.02 | .12 | .07 | .09 | .02 | .03 |
|  | 6 | -.03 | .07 | -.26 | .20 | .39 | 1 | .54 | .13 | .12 | -.04 | .06 | .21 | .15 | .20 | .27 | .09 | .11 | -.11 | <.01 | .14 | .30 | -.02 | .03 | .14 | .18 | -.15 | -.07 | -.13 | -.07 | -.07 | .07 | .12 | .03 | .07 | .12 |
|  | 7 | -.02 | .05 | -.22 | .23 | .41 | .58 | 1 | .12 | .38 | .05 | .36 | .26 | .39 | .24 | .48 | .17 | .35 | -.33 | .04 | .07 | .39 | -.16 | .14 | .08 | .40 | -.12 | -.03 | -.18 | -.02 | -.01 | -.04 | .23 | .11 | -.02 | -.01 |
|  | 8 | -.14 | <.01 | -.15 | .20 | .19 | .17 | .16 | 1 | .38 | .19 | .17 | .37 | .26 | .21 | .13 | .28 | .21 | -.19 | <.01 | -.08 | .18 | -.26 | .04 | .07 | -.05 | -.11 | -.01 | -.02 | <.01 | -.03 | .08 | -.09 | -.11 | .08 | .11 |
|  | 9 | -.11 | .06 | -.17 | .07 | .33 | .11 | -.01 | .19 | 1 | .14 | .56 | .34 | .49 | .24 | .44 | .30 | .29 | -.25 | .06 | -.10 | .42 | -.08 | .18 | -.02 | .11 | .01 | -.04 | -.19 | -.05 | -.05 | .13 | -.01 | <.01 | .07 | .01 |
|  | 10 | -.04 | -.04 | -.13 | .14 | .25 | .12 | .06 | .11 | .17 | 1 | .35 | .29 | .23 | .36 | .21 | .31 | .26 | -.25 | -.11 | -.01 | .08 | -.14 | -.07 | -.09 | -.10 | <.01 | .10 | .12 | .21 | .11 | .04 | -.19 | -.04 | .06 | .02 |
|  | 11 | -.02 | .07 | -.20 | .13 | .60 | .39 | .52 | .16 | .31 | .30 | 1 | .32 | .52 | .30 | .58 | .25 | .48 | -.28 | .16 | .06 | .48 | -.14 | .27 | -.06 | .09 | .14 | .12 | -.09 | .13 | .02 | .16 | .02 | .08 | .12 | .09 |
|  | 12 | -.04 | .21 | -.30 | .35 | .21 | .10 | .19 | .20 | .24 | .36 | .34 | 1 | .55 | .45 | .39 | .44 | .25 | -.26 | -.02 | -.01 | .26 | -.15 | .03 | .06 | .21 | .05 | .12 | -.09 | .06 | .03 | .05 | -.11 | -.01 | .09 | .20 |
|  | 13 | .05 | .13 | -.21 | .15 | .34 | .27 | .68 | .07 | .18 | .09 | .60 | .40 | 1 | .27 | .77 | .22 | .47 | -.27 | .20 | <.01 | .64 | -.01 | .35 | -.02 | .08 | .05 | .05 | -.19 | .11 | .01 | .12 | <.01 | -.01 | <.01 | .13 |
|  | 14 | .17 | -.13 | .03 | .18 | .18 | .03 | .15 | .18 | .16 | .21 | .21 | .33 | .24 | 1 | .38 | .33 | .17 | -.03 | .01 | .10 | .19 | -.01 | <.01 | -.06 | .13 | .10 | .16 | -.02 | -.04 | .04 | .08 | .01 | .16 | .15 | .05 |
|  | 15 | .06 | .08 | -.20 | .19 | .46 | .29 | .68 | .11 | .30 | .20 | .69 | .41 | .83 | .30 | 1 | .18 | .48 | -.21 | .19 | .11 | .68 | .09 | .31 | -.07 | .16 | -.01 | .07 | -.11 | .04 | -.02 | .07 | .12 | .13 | .02 | .10 |
|  | 16 | -.04 | .13 | -.25 | .32 | .26 | .29 | .26 | .12 | .11 | .29 | .26 | .37 | .28 | .23 | .25 | 1 | .21 | -.17 | -.01 | -.11 | .19 | -.19 | .06 | -.07 | .05 | .01 | .09 | -.01 | -.09 | <.01 | .04 | -.10 | -.11 | .04 | .10 |
|  | 17 | -.13 | .16 | -.23 | .15 | .49 | .41 | .52 | .18 | .13 | .23 | .66 | .32 | .42 | .11 | .50 | .25 | 1 | -.23 | .28 | .04 | .45 | -.12 | .33 | .01 | .06 | .05 | .07 | -.13 | .12 | -.06 | .13 | .06 | -.01 | .07 | .11 |
|  | 18 | .08 | -.15 | .17 | -.21 | -.14 | -.16 | -.21 | -.05 | .06 | -.05 | -.11 | -.04 | -.10 | .04 | -.02 | -.17 | -.21 | 1 | .10 | .34 | -.11 | .33 | -.06 | -.02 | -.19 | .20 | -.02 | .19 | -.07 | -.06 | .12 | .10 | .12 | .06 | .04 |
|  | 19 | <.01 | .13 | -.06 | .02 | .27 | .06 | .11 | -.05 | .27 | .07 | .28 | .20 | .22 | <.01 | .22 | -.03 | .18 | .17 | 1 | .03 | .41 | .11 | .77 | .08 | -.06 | .13 | .06 | -.07 | .11 | -.06 | .07 | -.01 | -.01 | -.01 | .07 |
|  | 20 | .24 | -.02 | .04 | -.26 | -.05 | -.12 | -.06 | <.01 | .03 | .02 | -.02 | .10 | .08 | .11 | .10 | -.18 | -.10 | .46 | .16 | 1 | .14 | .12 | -.12 | .13 | .02 | .13 | .07 | .01 | -.01 | -.03 | -.02 | .18 | .24 | .03 | <.01 |
|  | 21 | .03 | .08 | -.11 | -.06 | .12 | .07 | .01 | .01 | .14 | -.02 | .16 | .09 | .05 | .08 | .12 | -.06 | .25 | .02 | .42 | .07 | 1 | .13 | .53 | .09 | .19 | .07 | .06 | -.13 | -.04 | -.06 | .05 | .04 | -.03 | .03 | .10 |
|  | 22 | .03 | -.04 | .01 | -.03 | .03 | .07 | .02 | .08 | -.03 | .04 | .03 | -.01 | .06 | .07 | .07 | -.10 | -.01 | .03 | -.05 | .19 | .08 | 1 | .14 | <.01 | -.02 | -.03 | -.13 | -.01 | .06 | -.06 | .01 | .07 | .14 | -.10 | .01 |
|  | 23 | .03 | .21 | -.23 | .03 | .15 | .16 | .09 | -.05 | -.01 | -.04 | .21 | .03 | .08 | .07 | .12 | .08 | .38 | -.13 | .24 | -.08 | .78 | .12 | 1 | -.03 | .03 | .06 | -.01 | -.08 | .06 | -.05 | .13 | -.05 | -.14 | .03 | .08 |
|  | 24 | .01 | .16 | -.11 | <.01 | .14 | .13 | .15 | -.01 | .02 | .22 | .25 | .12 | .16 | -.02 | .17 | .19 | .26 | -.10 | .03 | -.23 | .12 | -.11 | .15 | 1 | .14 | <.01 | .03 | .14 | .01 | -.11 | -.04 | .10 | -.08 | -.04 | .01 |
|  | 25 | -.05 | .09 | -.22 | .05 | .16 | .19 | .22 | .07 | .05 | -.02 | .16 | .04 | .11 | -.05 | .07 | -.07 | .40 | -.16 | .12 | -.11 | .54 | -.07 | .44 | .11 | 1 | -.03 | .01 | -.12 | -.14 | <.01 | -.23 | .20 | -.01 | -.09 | -.07 |
|  | 26 | -.09 | -.09 | .03 | -.13 | -.08 | -.08 | .03 | .06 | .02 | -.06 | .06 | .04 | .09 | -.02 | .07 | -.20 | .02 | .30 | <.01 | .18 | -.08 | -.01 | -.15 | .02 | .03 | 1 | .50 | .09 | .08 | -.19 | .19 | -.21 | -.15 | .18 | .14 |
|  | 27 | -.04 | -.02 | .11 | -.13 | -.13 | -.15 | -.13 | -.06 | .05 | .05 | -.02 | .11 | .02 | .03 | .03 | -.15 | .02 | .21 | .04 | .16 | .08 | -.01 | -.03 | .11 | -.01 | .72 | 1 | .03 | .17 | -.07 | .02 | -.07 | -.02 | .19 | .18 |
|  | 28 | .01 | .04 | -.01 | -.10 | .07 | .04 | .21 | -.04 | -.09 | .07 | .17 | .05 | .11 | -.10 | .12 | -.08 | .20 | .13 | .06 | .18 | .04 | .15 | .05 | -.06 | .07 | .24 | .16 | 1 | .18 | -.01 | -.06 | .08 | .05 | -.07 | <.01 |
|  | 29 | .01 | .09 | .03 | .03 | .02 | .05 | .13 | -.02 | .10 | .02 | .16 | .06 | .16 | -.04 | .12 | -.07 | .06 | .14 | .19 | .05 | .05 | -.04 | .09 | -.06 | -.01 | .20 | .25 | .58 | 1 | -.11 | .16 | -.18 | -.05 | -.03 | .06 |
|  | 30 | .20 | .08 | -.01 | .02 | .03 | -.04 | .02 | .04 | -.03 | -.03 | .03 | -.03 | .05 | .06 | .06 | .03 | .05 | -.12 | -.14 | .13 | .05 | -.01 | .09 | -.13 | .13 | -.07 | -.07 | -.01 | -.06 | 1 | -.37 | .30 | .43 | -.12 | -.40 |
|  | 31 | -.23 | -.09 | .05 | -.05 | -.03 | -.11 | -.13 | -.15 | .02 | .03 | -.10 | <.01 | -.15 | -.22 | -.09 | -.07 | .01 | -.03 | .01 | -.14 | .02 | .01 | -.01 | .12 | .06 | .09 | .09 | -.04 | -.16 | -.40 | 1 | -.42 | -.33 | .37 | .32 |
|  | 32 | .24 | .04 | -.11 | .03 | -.01 | .02 | .09 | .22 | .06 | -.13 | .02 | <.01 | .16 | .21 | .09 | .07 | .01 | -.03 | -.03 | -.01 | -.03 | -.04 | -.05 | -.10 | -.02 | -.01 | -.14 | .04 | .11 | .27 | -.48 | 1 | .54 | -.24 | -.19 |
|  | 33 | .28 | .02 | -.21 | -.04 | .01 | .03 | .07 | .17 | .06 | -.15 | .01 | -.06 | .09 | .09 | .02 | .02 | -.02 | -.05 | -.05 | -.01 | .10 | -.02 | .13 | -.08 | .10 | -.14 | -.21 | -.06 | .02 | .29 | -.34 | .58 | 1 | -.06 | -.22 |
|  | 34 | -.13 | -.13 | .22 | -.08 | -.03 | <.01 | -.22 | -.10 | -.03 | .01 | -.18 | -.10 | -.24 | -.15 | -.20 | -.10 | -.19 | .10 | .02 | -.14 | -.02 | .07 | -.01 | .05 | -.05 | -.02 | -.10 | .05 | -.12 | -.26 | .32 | -.30 | -.16 | 1 | .36 |
|  | 35 | -.09 | -.02 | .02 | -.15 | -.19 | -.03 | -.04 | -.02 | -.05 | -.02 | -.16 | -.11 | -.02 | -.04 | -.06 | -.09 | -.02 | .13 | .04 | .02 | .07 | .03 | .06 | .08 | -.03 | .05 | .17 | .02 | <.01 | -.35 | .29 | -.09 | -.13 | .26 | 1 |

*Noot.* Voor continue en/of dichotome variabelen zijn Pearson correlaties berekend. Bij ordinale variabelen of een combinatie van dichotome variabelen zijn Spearman correlaties berekend. Boven de diagonaal: de correlaties in voorjaar 2019; onder de diagonaal: de correlaties in voorjaar 2020. Donkergroen = positieve correlatie en *p* <.001; lichtgroen = positieve correlatie en *p* <.05; rood = negatieve correlatie en *p* <.001; oranje = negatieve correlatie en *p* <.05; lichtgrijs = niet significant.

Demografische gegevens

1. Gender (dichotoom; 1 = jongen; 2 = meisje)
2. Leeftijd
3. Opleidingsniveau (dichotoom; 1 = vmbo; 2 = havo of vwo)

Drinken

1. Frisdrank suiker thuis
2. Frisdrank suiker buitenshuis
3. Energiedrankjes thuis
4. Energiedrankjes buitenshuis
5. Zoete melkdrankjes thuis
6. Zoete melkdrankjes buitenshuis

Eten

1. Gebak, cake, grote koeken thuis
2. Gebak, cake, grote koeken buitenshuis
3. Candybars thuis
4. Candybars buitenshuis
5. Chocolade thuis
6. Chocolade buitenshuis
7. Warme snacks thuis
8. Warme snacks buitenshuis
9. Fruit thuis
10. Fruit buitenshuis
11. Rauwe groenten thuis
12. Rauwe groenten buitenshuis
13. Warme groenten thuis
14. Warme groenten buitenshuis

Leefstijl

1. Alcoholgebruik (dichotoom; 0 = niet wekelijks; 1 = wekelijks)
2. Roken (dichotoom; 0 = niet wekelijks; 1 = wekelijks)
3. Zware fysieke inspanning, dagen per week
4. Zware fysieke inspanning, tijd per keer (ordinaal; 7 categorieën van 0 = “0 uur” tot 7 = “3 uur or meer”)
5. Matige fysieke inspanning, dagen per week
6. Matige fysieke inspanning, tijd per keer (ordinaal; 7 categorieën van 0 = “0 uur” tot 7 = “3 uur or meer”)

Sociaal-emotionele gezondheid

1. Eenzaamheid
2. Geluk
3. Stress thuis
4. Stress door school
5. Relatietevredenheid met ouders
6. Relatietevredenheid met beste vriend
